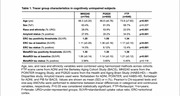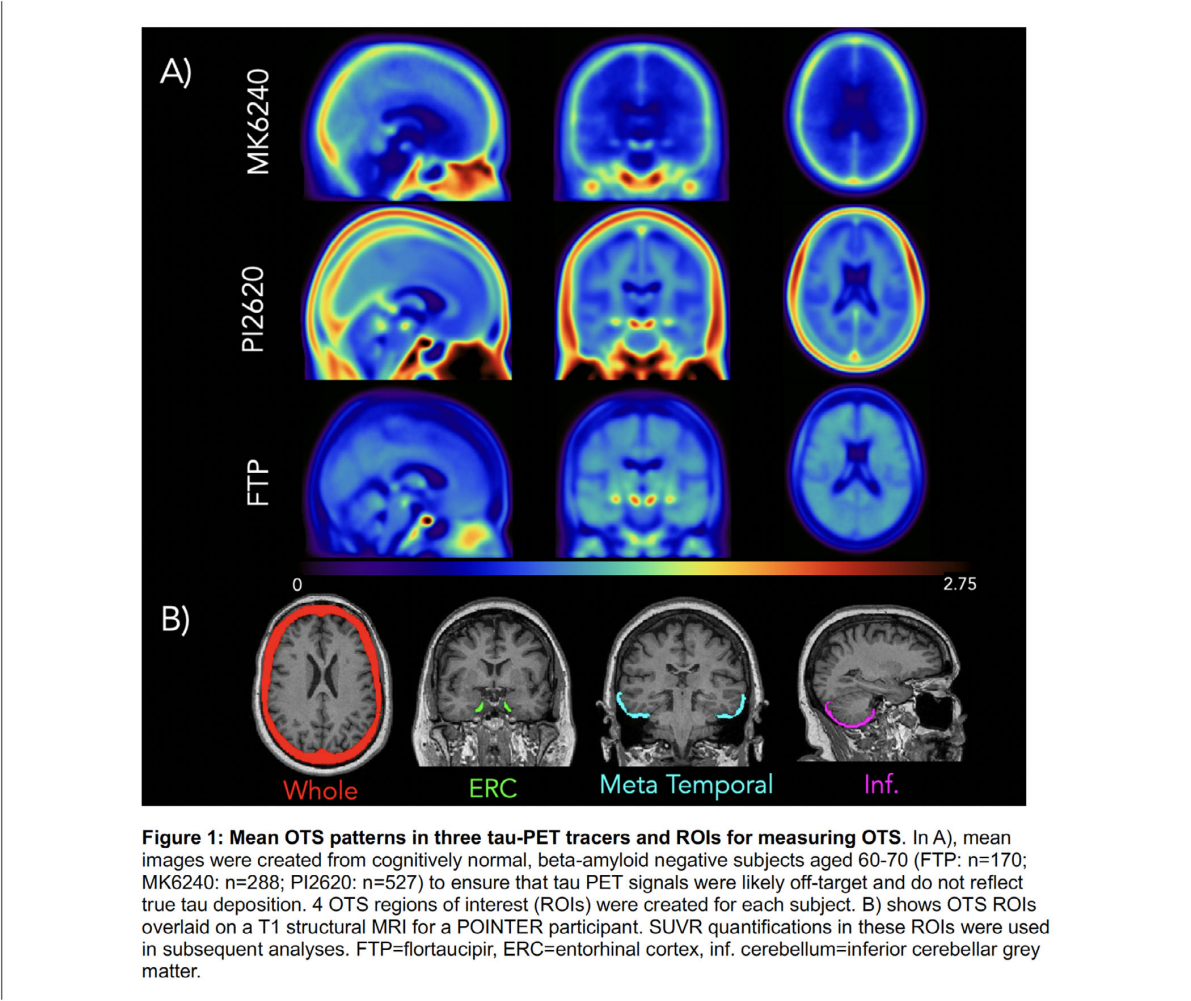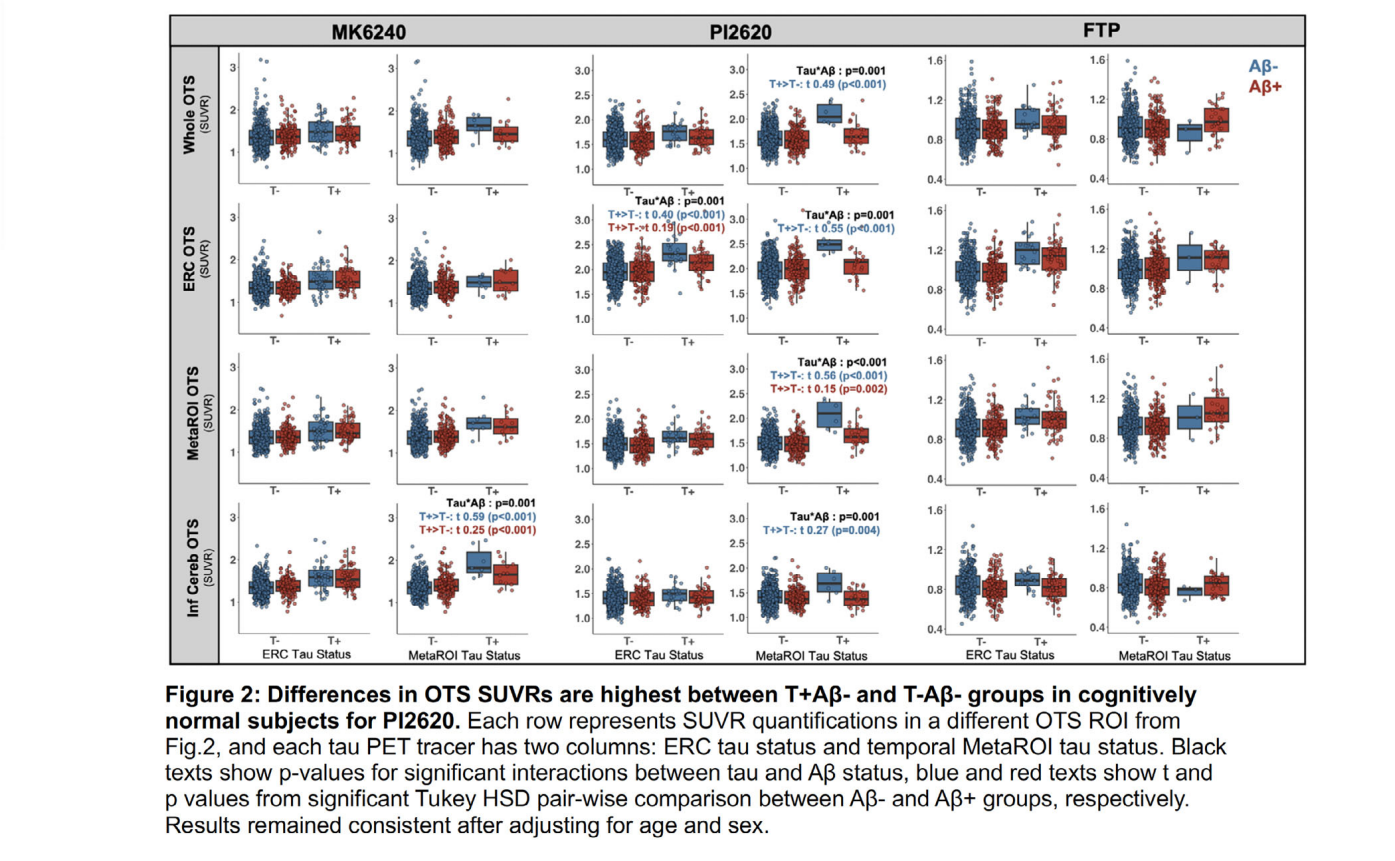# Tau PET positivity rates in relation to meningeal off‐target signal MK6240, PI2620, and flortaucipir scans

**DOI:** 10.1002/alz70856_100456

**Published:** 2025-12-25

**Authors:** Yishu Chao, Trevor A Chadwick, Karly Cody, Elizabeth C. Mormino, Suzanne L. Baker, Susan M. Landau, William J. Jagust, Theresa M. Harrison

**Affiliations:** ^1^ University of California, Berkeley, Berkeley, CA, USA; ^2^ Department of Neurology and Neurological Sciences, Stanford University, Stanford, CA, USA; ^3^ Lawrence Berkeley National Laboratory, Berkeley, CA, USA; ^4^ Neuroscience Department, University of California, Berkeley, Berkeley, CA, USA

## Abstract

**Background:**

We performed this study to investigate whether and how off‐target signal (OTS) associated with tau PET ligands affects the rates of tau positivity when using quantitative approaches to disease staging.

**Method:**

MK6240, PI2620, and FTP PET scans and contemporaneous structural MRI scans from older adults aged >55 were processed through harmonized pipelines. All tau PET scans were normalized by inferior cerebellum. Scans from cognitively unimpaired (CU), Ab‐ participants aged 60‐70yrs were averaged in template space to visualize OTS patterns for each tracer. Previously developed OTS ROIs were applied in all participants: a whole OTS ROI (meninges and other OTS hotspots) along with OTS ROIs adjacent to entorhinal cortex (ERC), temporal MetaROI and inferior cerebellum (Figure 1). Amyloid status was defined using global beta‐amyloid (Ab) SUVRs. Tracer‐specific entorhinal cortex (ERC) and MetaROI tau positivity thresholds were derived from gaussian mixture models with all participants >55 years. Two‐way ANOVAs were used to test for interaction between tau and amyloid status on OTS SUVRs.

**Result:**

2200 CU older adults across 3 tracer groups were included (Table 1). Template‐space mean images showed distinct OTS patterns for each tracer (Figure 1) — MK6240 had meningeal binding but low signal everywhere else; PI2620 had skull, meningeal, and white matter OTS; and while FTP had little meningeal OTS, it showed elevated signal in the basal ganglia and white matter. For PI2620, two‐way ANOVAs revealed interaction effects between MetaROI tau status and Ab status in all 4 OTS ROIs and between ERC tau status and Ab status on ERC‐adjacent OTS SUVRs (Figure 2). Post‐hoc pairwise comparisons showed that these interactions were either entirely driven by the Ab‐ subgroup, or the differences in OTS SUVRs between T+ and T‐ groups were greater in Ab‐ than in Ab+ groups. Similar interactions were largely not observed in MK6240 or FTP.

**Conclusion:**

Elevated OTS was observed in the T+Ab‐ group in PI2620 across all OTS ROIs. Since CU Ab‐ subjects are not expected to have above‐threshold tau burden, OTS may be spilling into target regions resulting in false positives. Next steps include visual inspection of T+Ab‐ scans to determine OTS‐related false tau positivity rate.